# High-Resolution Mechanical Imaging of Glioblastoma by Multifrequency Magnetic Resonance Elastography

**DOI:** 10.1371/journal.pone.0110588

**Published:** 2014-10-22

**Authors:** Kaspar-Josche Streitberger, Martin Reiss-Zimmermann, Florian Baptist Freimann, Simon Bayerl, Jing Guo, Felix Arlt, Jens Wuerfel, Jürgen Braun, Karl-Titus Hoffmann, Ingolf Sack

**Affiliations:** 1 Department of Radiology, Charité - Universitätsmedizin Berlin, Berlin, Germany; 2 Department of Neuroradiology, Universitätsmedizin Leipzig, Leipzig, Germany; 3 Department of Neurosurgery, Universitätsmedizin Göttingen, Göttingen, Germany; 4 Department of Neurosurgery, Charité - Universitätsmedizin Berlin, Berlin, Germany; 5 Department of Neurosurgery, Universitätsmedizin Leipzig, Leipzig, Germany; 6 Institute of Neuroradiology, Universitätsmedizin Göttingen, Göttingen, Germany; 7 NeuroCure, Charité - Universitätsmedizin Berlin, Berlin, Germany; 8 Institute of Medical Informatics, Charité - Universitätsmedizin Berlin, Berlin, Germany; University of Maryland, College Park, United States of America

## Abstract

**Objective:**

To generate high-resolution maps of the viscoelastic properties of human brain parenchyma for presurgical quantitative assessment in glioblastoma (GB).

**Methods:**

Twenty-two GB patients underwent routine presurgical work-up supplemented by additional multifrequency magnetic resonance elastography. Two three-dimensional viscoelastic parameter maps, magnitude |G*|, and phase angle *φ* of the complex shear modulus were reconstructed by inversion of full wave field data in 2-mm isotropic resolution at seven harmonic drive frequencies ranging from 30 to 60 Hz.

**Results:**

Mechanical brain maps confirmed that GB are composed of stiff and soft compartments, resulting in high intratumor heterogeneity. GB could be easily differentiated from healthy reference tissue by their reduced viscous behavior quantified by φ (0.37±0.08 vs. 0.58±0.07). |G*|, which in solids more relates to the material's stiffness, was significantly reduced in GB with a mean value of 1.32±0.26 kPa compared to 1.54±0.27 kPa in healthy tissue (P = 0.001). However, some GB (5 of 22) showed increased stiffness.

**Conclusion:**

GB are generally less viscous and softer than healthy brain parenchyma. Unrelated to the morphology-based contrast of standard magnetic resonance imaging, elastography provides an entirely new neuroradiological marker and contrast related to the biomechanical properties of tumors.

## Introduction

Despite recent advances in operative and postoperative treatment, glioblastoma (GB) still remains one of the most malicious and aggressive and malignant forms of cancer [1], [2]. It accounts for >50% of all primary neuroepithelial tumors and approximately 20% of all brain tumors [3]. In developed countries, the incidence of GB is 3.5 per 100,000 population per year [4], [5], [6]. The term GB was first introduced in 1926 by Percival Bailey and Harvey Cushing and refers to the cellular origin from glioblasts and the histological heterogeneity of this brain tumor [7]. The classification of the World Health Organization (WHO) ranks GB as a grade IV tumor due to its histological characteristics with aggressive and infiltrative growth and overall poor prognosis [8]. Despite aggressive surgical resection, radiotherapy, and chemotherapy the prognosis for patients newly diagnosed with GB remains poor with a 2-year survival rate of only 13–26% and a mean survival time of 12–15 months [2].

Neuroradiological assessment of GB and differentiation from solitary intracranial metastases or lymphomas is challenging due to the tumor's heterogeneous composition resulting from the presence of cysts, necrosis, and hemorrhage [9], [10]. As a consequence, diagnostic biopsy remains inevitable for a definitive diagnosis despite possible complications [11]. Advanced magnetic resonance imaging (MRI) methods such as diffusion tensor imaging provide structural information related to water mobility in white matter tracts but cannot reveal the consistency and mechanical constitution of biological tissue[12], [13]. Targeting the mechanical properties of GB potentially provides information about the tumor's structural heterogeneity as well as its perifocal tissue infiltration which is of relevance for diagnosis, therapy planning and treatment monitoring.

Today, the viscoelastic properties of the brain can be assessed noninvasively by magnetic resonance elastography (MRE) [14]. Combining time-harmonic vibrations in the low audible range with motion-sensitive MRI, cerebral MRE [15], [16], [17] has proven sensitive to mechanostructural changes in the human brain associated with aging [18], [19] and diseases [20], [21], [22], [23], [24], [25]. Recent results in mouse models suggest that cerebral MRE is sensitive to demyelination, inflammation, and extracellular matrix alterations [26], [27], [28] and may thus provide new information about structural changes of cerebral tissue in brain tumors. Indeed, initial findings in different intracranial tumor entities [29] including meningeomas [30], [31] indicate the feasibility of MRE for the presurgical assessment of neuronal tumor consistency.

However, previous studies were limited by low spatial resolution of the mechanical parameter maps achievable by MRE at a single harmonic drive frequency. Recent advances in fast image acquisition schemes [32], [33] and wave field reconstruction methods [34], [35], [36], [37] enabled us to acquire 3D wave fields at multiple vibration frequencies, generating cerebral MRE maps with a spatial resolution comparable to that of normal MRI [38], [39].

In this study we applied multifrequency MRE (MMRE) for in vivo high resolution mechanical imaging of GB tissue including perifocal brain parenchyma as a surrogate for infiltrative tumor growth, and distant tumor edema in comparison to tumor and reference tissue. The lack of clearly delineable solid-type tissue within many tumors led us to further specify regions with homogeneous appearance in standard T2- and contrast-enhanced T1-weighted MR images.

By these regions, we address for the first time whether high-resolution multifrequency MRE can measure the heterogeneity of GB tissue mechanics which is likely linked to the well known microstructural heterogeneity of GB including neovascularisation and central hemorrhage. Despite the fact that any spatial averaging over-simplifies the tumor's intrinsic heterogeneity, we will tabulate mechanical property values of GB as a starting point for quantification, diagnostic assessment and therapy planning of GB by MRE.

## Methods

Twenty-two patients with histologically proven GB (mean age 64.5±15.1 years; 10 women) were included in this study. Each patient underwent clinical MRI and MRE prior to further diagnostic (e.g., biopsy) or therapeutic procedures. In addition to MRE, a clinical protocol including T1-, T2-, and proton-density-weighted sequences was applied before and after administration of gadolinium-based contrast agent for further evaluation of the lesion. Imaging slices for MRE were positioned in transverse orientation according to tumor site.

The study was approved by the ethics committee of the Charité - Universitätsmedizin Berlin (EA1/261/12). All patients gave informed written consent prior to MRE.

### MMRE

A custom-designed nonmagnetic driver based on piezoelectric ceramics [33] was mounted at the end of the patient table. The vibrations were transmitted by a carbon fiber rod connected to a custom-designed head cradle located inside the head coil. Eleven of the experiments were performed on a 1.5T MRI scanner (Magnetom Sonata; Siemens Erlangen, Germany), the remaining eleven experiments were carried out on a 3T MRI scanner (Trio; Siemens Erlangen, Germany) using a 4-channel (1.5T) and a 12-channel (3T) head coil. The imaging sequence parameters for both systems are listed below. After acquisition of a localizer and a 3D T1-weighted sequence for anatomical reference, a single-shot spin-echo echo-planar imaging sequence with trapezoidal flow-compensated motion-encoding gradients (MEG), consecutively applied along all three axes of the scanner coordinate system, was used for rapid motion field acquisition [40]. A custom-made head cradle was used to generate mechanical shear waves inside the brain by inducing a gentle nodding motion of the head. To allow the mechanical waves to propagate into the tissue, the vibration was initiated through a trigger pulse by the scanner at least 100 ms before the start of the MEG. The vibration frequencies (*f*) used in this experiment were 30, 35, 40, 45, 50, 55, and 60 Hz. The trigger pulse was delayed in consecutive time-resolved scans by increments of 1/(8*f), yielding 8 dynamics of a wave cycle. For 10 (1.5T) and 15 (3T) adjacent slices of 2×2×2 mm^3^ resolution, 7 frequencies, 8 wave dynamics, and 3 MEG directions were applied. Further imaging parameters were: repetition time (TR) 2400 ms (1.5T) and 3000 ms (3T); echo time (TE) 99 ms (1.5T) and 71ms (3T); field of view (FoV) 192×176 mm^2^ (1.5T) and 250×188 mm^2^ (3T); matrix size 88×96 (1.5T) and 128×64 (3T); no parallel imaging at 1.5T, GRAPPA factor of 2 at 3T; MEG frequencies (number of MEG periods): 25 (1), 26 (1), 30 (1), 50 (2), 50 (2), 50 (2), 54 (2) Hz corresponding to 30, 35, 40, 45 ,50, 55, 60 Hz vibration frequency, respectively (note: MEG frequency and period number were chosen to accomplish the highest encoding efficiency according to the principle of fractional motion encoding [41] and given by equation 4e in [40]); MEG amplitude 30 mT/m (1.5T) and 35 mT/m (3T); ∼1 min scan time for each frequency, resulting in a total acquisition time of ∼7 min for a full multislice MMRE examination.

### Data Postprocessing

Wave image postprocessing followed the strategy outlined in [42]. In brief: First, the complex MR images were smoothed using a 2D Gaussian filter with a kernel of 5 pixel edge length and sigma  = 0.65 for noise reduction. Subsequent gradient-based unwrapping was performed as described by [43]. First-order in-plane derivatives along the image coordinate axes 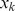 (*k* = 1,2) (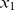 is the phase-encoding direction and 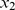 the read-out direction) of the spin phase 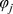 (*j* = 1,2,3) were calculated by:
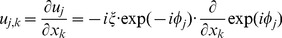
(1)


Factor *ξ* scales the spin phase 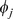 to the physical displacement component 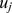 (in meters) according to [40]. After Fourier transformation in time, equation (1) yields six complex-valued strain images 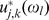 at angular drive frequency 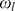, resulting in a total of 42 images at each slice invoked by the reconstruction algorithm. These images were further smoothed by a 2D Butterworth lowpass filter with a threshold of 100 m^−1^. Low wave numbers as resulted by compression waves were considered sufficiently suppressed by the derivative operators. Other than in previous work [33], [38], [39], we abandoned curl components for wave inversion since interslice phase artifacts, as addressed by [44], impair the derivative operator in the 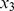 direction. Instead of three curl components for each frequency, six independent strain wave images were obtained using eq.(1). All six images were used for stabilizing the wave inversion as described in the following. This strategy is further outlined in [42].

We applied multifrequency dual elasto visco (MDEV) inversion [33], [38], [39]. This algorithm provides two independent mechanical constants, |G*| and *φ*, corresponding to the magnitude and phase of the complex shear modulus G*. |G*| provides an indication of the softness or firmness of the tissue while *φ* provides an indication of the viscous, i.e. dissipative, tissue properties. Of note, both parameters are model-free and provide just another representation of the storage and loss modulus usually parameterized in MRE. It is well known that the loss and storage modulus of brain tissue display dispersion within the examined frequency range [18]. In MDEV inversion-based MRE, we sacrifice the information provided by frequency-resolved complex shear moduli for generating spatially highly resolved maps of |G*| and *φ* [33]. As a result, |G*| and *φ* refer to the amplitude and phase angle of the oscillatory response to a harmonic stress, respectively. The effective harmonic frequency of |G*| and *φ* is given by the mean of all vibration frequencies weighted by the wave amplitudes they produced.

Accounting for complex-valued shear strain images 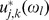 and making the usual assumptions in MRE such as homogeneity, linear viscoelasticity, and isotropy, we obtain the following inversion equations:
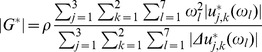
(2a)

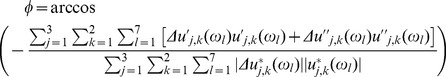
(2b)


Given that 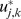 represents in-plane strain components and Δ denotes the 2D-Laplacian, our inversion is entirely 2D-based. By these equations we implemented the method proposed in [39] where data and data derivatives are projected onto the ones vector instead of derivative vector as done in classical least squares solutions of the wave equation [45]. The ones-vector model refers to the almost trivial regression of repeated measurements by computing the observational average (see eqs. 2.81 and 2.82 in [46]).

### Morphological Tumor Assessment

**Figure 1:**
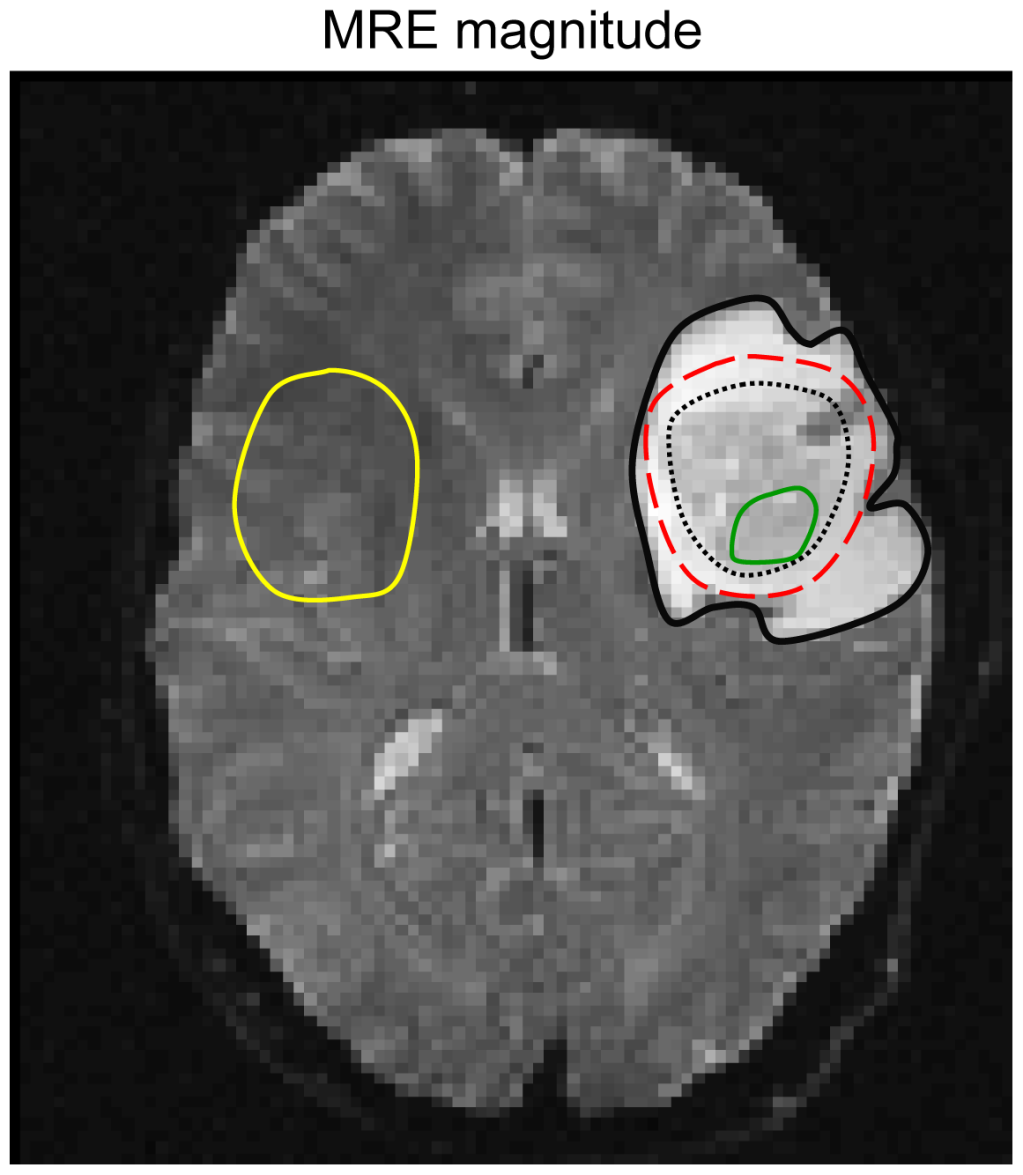
Example illustrating how the regions of interest (ROI) are defined one slice of the MRE magnitude image of patient #10: healthy tissue (yellow), tumor (black dotted line), perifocal margin (red dashed line), and edema (black solid line). The green line demarcates the region chosen for homogeneous appearing matter (HAM).

MRI-based tumor morphology was classified and graded using T2-weighted (T2w) images as well as contrast-enhanced T1-weighted (T1w) images. These images were used to assess tumor morphology including e.g. the presence of cysts, homogenous appearing tumor portion, and necrosis. The tumor volume was calculated using the OsiriX-Imaging software (Geneva, Switzerland) and the MiaLite plugin (SPIE medical imaging 2011, Lake Buena Vista, Florida, USA) by defining contrast enhancing tissue on T1-weighted images. Assessment of tumor morphology and tumor size was performed by experienced neuroradiologists (M.R.-Z., K.-T.H.). Regions of interest (ROI) were manually selected on the basis of image contrast in the MRE magnitude images for the tumor, the edema, and healthy tissue (normal appearing white matter) in a corresponding contralateral region as demonstrated in Figure 1. The selection was done by one observer experienced in neurological MRI and MRE (K.-J.S.) and further confirmed or revised by two experienced neuroradiologists (M.R.-Z., J.W.). Standard deviations of |G*| and φ were calculated for the tumor ROI to indicate the heterogeneity of the tumor's viscoelastic properties. Additionally, we selected a region of apparently high homogeneity within the tumor region based on morphological MRI (HAM –homogeneous appearing matter) to further compartmentalize the tumor and therewith to address the intra-tumor heterogeneity. In order to study the effect of uncertainties in tumor margins, invasion of surrounding tissue, and partial volume effects, a perifocal ROI was automatically defined by dilatation of the tumor ROI by three pixels minus the tumor region, yielding a small ring around the tumor as illustrated in Figure 1. All regions were single objects delineated in multiple slices. Similarly, tumor volume determination was based on three dimensions, i.e., area analysis was performed on consecutive sections in adjacent slices.

### Statistical Analysis

The results are tabulated as arithmetic mean ± standard deviation. The regional differences between tumor, homogenous appearing matter, perifocal region, edema, and corresponding healthy tissue in |G*| and *φ* were analyzed by two-tailed paired Student's t-test and Wilcoxon signed-rank test. The Gaussian distribution of the data was tested using the Lilliefors test and the Shaprio-Wilk test. Possible correlations between age and the viscoelastic properties of our regions of interest as well as between tumor volume and viscoelastic properties of the tumor were tested using linear and rank correlation. A P-value <0.05 was considered statistically significant. All calculations were performed using the MATLAB Statistics Toolbox (MathWorks, Natick, Massachusetts, USA).

## Results

**Figure 2:**
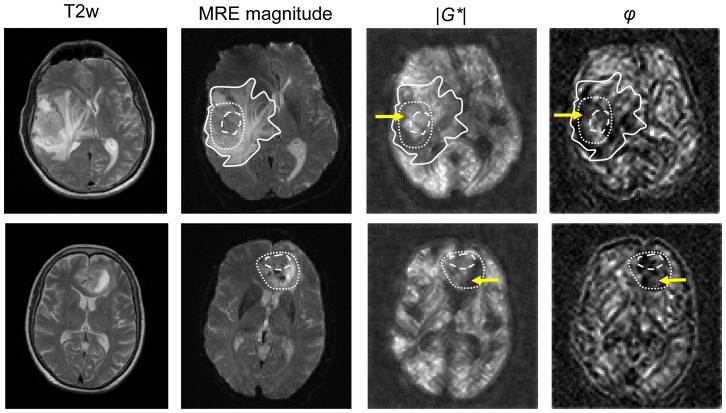
Anatomical T2-weighted images (T2w), MRE magnitude images, and 3DMMRE parameter maps (|G*| and *φ*) of 2 GB patients (upper row: patient #15, bottom row: patient #14, corresponding to the tables). The selected regions of tumor (dotted lines) and edema (solid line in #15) were used for the parameter evaluation as given in Table 2. The region of HAM is indicated by the dashed line. The yellow arrows indicate compartments of soft tissue properties (low |G*|) but different dissipative behavior (*φ*) in both tumors. |G*| was scaled from 0 to 3 kPa, φ was scaled from 0 to 2.5 rad.

The results regarding tumor morphology are summarized in Table 1. Figure 2 presents two cases with stiff and soft spatially averaged properties within the tumor ROI (On average, patient #15 [upper row] has an approximately 33% stiffer tumor compared to surrounding tissue. Vice versa, patient #14 has approximately 33% reduced tumor stiffness [bottom row]). In the upper row, an extended edematous region is visible. Both types of GB present compartments of soft properties with distinct dissipative behavior. While the soft GB compartment in patient #15 has low *φ* values, the soft GB region in #14 presents with higher dissipative properties, indicating necrotic liquefaction (yellow arrows). The heterogeneity of mechanical tissue properties is further reflected by group-averaged values given for GB, HAM, perifocal region, edema, and contralateral healthy reference tissue in Table 2. Intrinsic tumor heterogeneity is indicated by the standard deviation values for |G*| and φ within the tumor regions.

**Table 1:** Patient data, morphological classification on the basis of conventional MRI, size, and location of all tumors included in this study.

Pat. #	Sex	Age yrs.	Tumor size mm^3^	Morphology	Morph. staging	Location	Hemisphere	MRI field strength in T
1	m	55	5.9	mostly homogenous appearing tumor	1	parietal	left	3
2	m	61	83.0	mostly homogenous appearing tumor, few cysts	1	temporal	left	3
3	m	75	10.0	homogenous appearing tumor with central necrosis	1	parietal	left	3
4	f	60	22.6	large central necrosis with haemorrhage	3	parieto-occipital	left	3
5	m	42	37.8	mostly homogenous tumor with cysts	1	temporo-occipital	right	3
6	m	79	67.9	mostly homogenous tumor	1	corpus callosum	bilateral	3
7	f	86	17.9	many small cysts, no hemorrhage	2	frontal	right	3
8	m	80	46.5	large central hemorrhage	3	frontal	right	3
9	f	72	39.0	homogenous appearing tumor with small cystic fraction	2	parietal+ventricle	right	3
10	f	54	14.0	indistinct tumor edge with necrotic fraction, only a few cysts and homogenous portion	3	fronto-temporal	left	1.5
11	f	53	9.7	mostly homogenous appearing tumor with central necrosis, no cysts, no hemorrhage	1	parieto-occipital	left	1.5
12	f	65	30.8	diffuse tumor with few homogenous appearing fractions	3	parietal	left	1.5
13	m	76	16.8	mostly homogenous tumor with discrete central necrosis, no cysts	1	occipital, CC	right	1.5
14	f	72	22.1	cystic tumor, no hemorrhage	2	frontal	bilateral (l>r)	1.5
15	m	61	31.6	tumor with central necrosis and hemorrhage, only small homogenous appearing portion	3	temporal	right	1.5
16	f	61	25.1	mostly homogenous appearing tumor, few cysts, discrete haemorrhage	1	occipital	right	1.5
17	m	18	99.8	mostly cystic, homogenous appering tumor rostrally, discrete haemorrhage	2	frontal	right	1.5
18	m	59	140	mostly cystic, no hemorrhage	2	temporo-parietal	links	1.5
19	m	75	20.2	mostly homogenous appearing tumor, few cysts	1	fronto-temporal	links	1.5
20	m	62	17.8	mostly homogenous appearing tumor, few cysts	1	temporal	rechts	1.5
21	f	75	7.7	cystic tumor with central necrosis	3	parietal	left	3
22	f	78	33.2	cystic tumor with central necrosis	3	frontal	right	3

Morphological staging was performed according to the predominant tissue type of homogenous appearing mass ( = 1), cysts ( = 2), and necrosis/hemorrhage( = 3).

CC  =  corpus callosum.

**Table 2:** |G*| and *φ* values of the selected regions of interest (ROIs) as well as the parameter ratios of tumor ROI to healthy tissue ROI (normal appearing white matter) of the 22 patients included in the study.

Pat. #	|G*|_tumor_ kPa	|G*|_HAM_ kPa	|G*|_healthy_ kPa	|G*|_perifocal_ kPa	|G*|_edema_ kPa	φ_tumor_ rad	φ_HAM_ rad	φ_healthy_ rad	φ_perifocal_ rad	φ_edema_ rad	|G*|_tumor/_|G*|_healthy_	φ_tumor/_φ_healthy_
1	1.83 (0.308)	1.75	1.70	1.91	2.12	0.51 (0.32)	0.31	0.66	0.52	0.43	1.08	0.78
2	1.24 (0.471)	1.23	1.54	1.03	1.15	0.38 (0.28)	0.28	0.58	0.47	0.39	0.80	0.66
3	1.35 (0.431)	1.31	1.24	1.29	1.77	0.30 (0.24)	0.47	0.47	0.31	0.39	1.09	0.64
4	1.32 (0.724)	1.02	1.87	1.46	2.19	0.45 (0.31)	0.26	0.50	0.45	0.47	0.71	0.90
5	1.29 (0.541)	1.61	1.74	1.42	1.66	0.38 (0.29)	0.39	0.60	0.44	0.33	0.74	0.63
6	1.52 (0.464)	1.84	1.65	1.38	1.53	0.48 (0.25)	0.44	0.68	0.46	0.30	0.92	0.71
7	1.55 (0.446)	2.07	1.73	2.03	no edema	0.46 (0.23)	0.73	0.57	0.53	no edema	0.90	0.81
8	0.97 (0.420)	1.04	1.66	1.41	1.90	0.34 (0.28)	0.61	0.58	0.43	0.51	0.59	0.59
9	1.47 (0.539)	1.89	1.68	1.33	1.27	0.44 (0.25)	0.42	0.60	0.43	0.28	0.88	0.73
10	1.57 (0.320)	1.50	1.37	1.73	1.83	0.26 (0.15)	0.27	0.57	0.36	0.36	1.15	0.45
11	1.39 (0.548)	1.18	1.51	1.12	1.83	0.39 (0.25)	0.26	0.63	0.38	0.53	0.92	0.61
12	0.85 (0.358)	0.99	1.11	1.27	no edema	0.35 (0.23)	0.47	0.41	0.42	no edema	0.76	0.85
13	1.34 (0.359)	1.19	1.63	1.88	1.55	0.17 (0.15)	0.56	0.58	0.32	0.29	0.82	0.29
14	1.01 (0.323)	1.26	1.53	1.33	no edema	0.36 (0.23)	0.3	0.58	0.52	no edema	0.66	0.63
15	1.32 (0.395)	1.51	0.99	1.09	1.23	0.43 (0.24)	0.67	0.59	0.31	0.38	1.34	0.73
16	1.08 (0.261)	1.24	1.26	1.13	1.22	0.34 (0.21)	0.38	0.50	0.29	0.25	0.86	0.67
17	0.95 (0.349)	1.06	1.32	0.89	no edema	0.39 (0.27)	0.2	0.73	0.30	no edema	0.72	0.54
18	1.14 (0.415)	1.33	1.27	1.34	1.39	0.37 (0.19)	0.46	0.56	0.55	0.33	0.9	0.66
19	1.79 (0.287)	1.95	1.69	1.45	no edema	0.37 (0.25)	0.32	0.61	0.47	no edema	1.06	0.61
20	1.22 (0.154)	1.25	1.35	1.04	no edema	0.43 (0.21)	0.35	0.55	0.59	no edema	0.91	0.79
21	1.43 (0.299)	1.86	2.08	1.39	1.82	0.36 (0.2)	0.41	0.55	0.42	0.35	0.69	0.65
22	1.49 (0.321)	1.86	1.91	1.44	1.53	0.34 (0.2)	0.37	0.61	0.44	0.35	0.77	0.55
Mean	1.32	1.45	1.54	1.38	1.63	0.37	0.41	0.58	0.43	0.37	0.88	0.66
SD	0.26	0.34	0.27	0.29	0.32	0.08	0.14	0.07	0.09	0.08	0.19	0.15

The standard deviations of |G*| and *φ* within the tumor indicating the tumor heterogeneity are given in the brackets.

tumor: region of GB, HAM: region of homogeneous appearing matter, healthy: region of normal appearing white matter, perifocal: ring of tissue obtained by dilatation of the tumor region, edema: region of edematous tissue.

The mean |G*| value for all tumors was 1.32±0.26 kPa ranging, from 0.85 kPa (softest tumor) to 1.83 kPa (stiffest tumor). On average, healthy tissue was significantly stiffer than GB with a mean value of 1.54±0.27 kPa and a range of 0.99–2.08 kPa (P = 0.001); however, in a group of 5 tumors, higher |G*| values compared to reference tissue were observed (P = 0.015).

Mean *φ* was 0.37±0.08 for the tumor tissue and 0.58±0.07 for the corresponding healthy tissue. Interestingly, this reduction in *φ* was seen in all tumors (P = 2.9×10^−10^) regardless of their |G*| values, which suggests less dissipative (viscous) GB properties compared to healthy tissue.

Within the tumor, homogenous appearing matter showed a higher |G*| compared to full GB regions (P = 0.012) without different appearance to healthy tissue (P = 0.228), suggesting that HAM consists of less affected tissue than the remaining GB. Nevertheless, *φ*_HAM_ was still lower than *φ*_healthy_ (P = 0.00013) without significant difference to *φ*_tumor_ (P = 0.40). This high sensitivity of *φ* to GB is further represented by the normalized ratios *φ*_tumor_/*φ*_healthy_ which are below 1 in all patients indicating the viability of *φ* as diagnostic biomarker.

**Figure 3:**
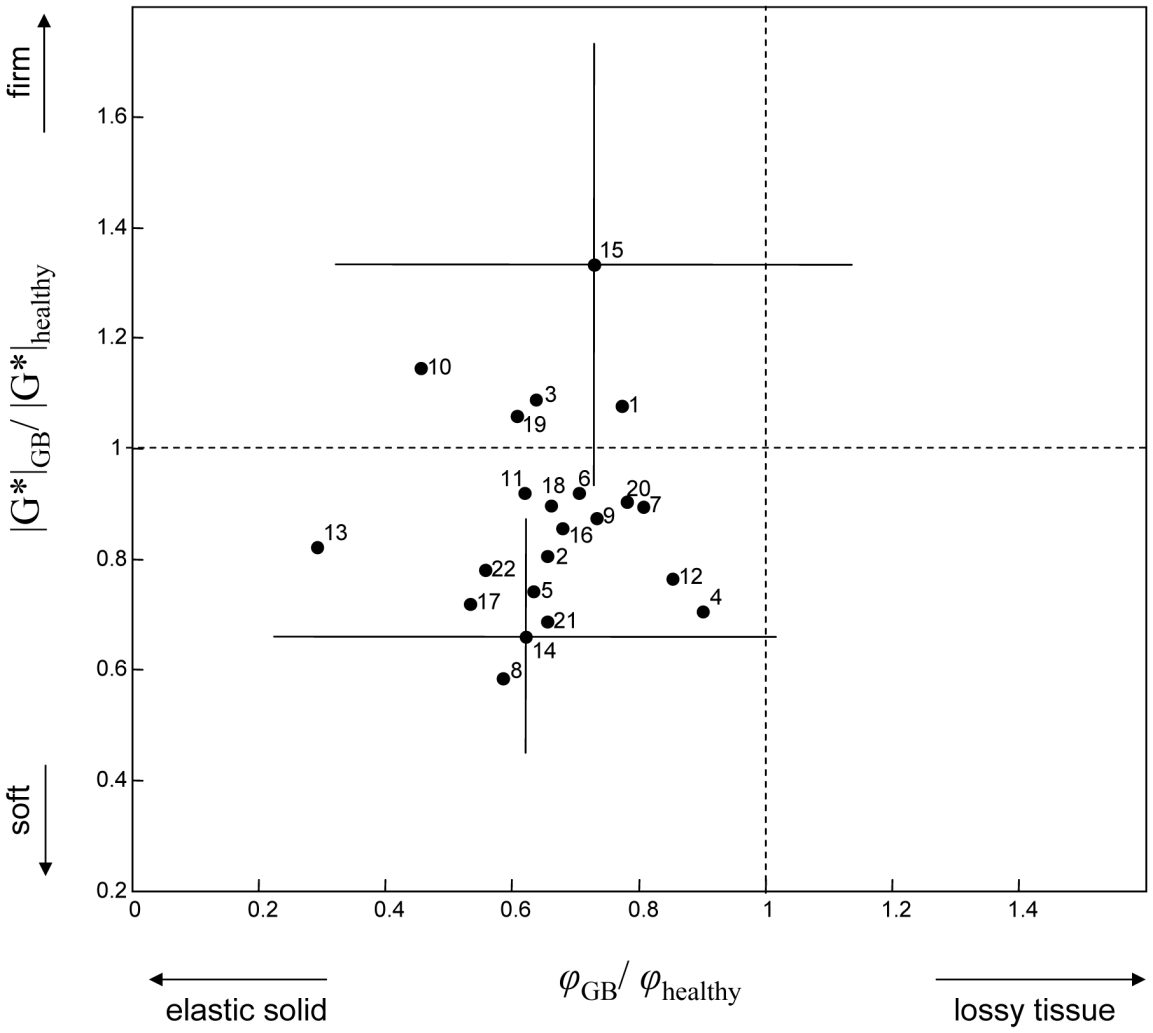
Viscoelastic properties of GB based on the parameter ratios of |G*| and φ between tumor and healthy reference tissue (ref). Numbers correspond to the list of tumors in tables 1 and 2. For illustration purposes, standard deviations (SD) indicating intratumoral heterogeneity are shown for the two tumors presented in figure 2 (solid lines in 14, and 15). All SD values are given in table 2.

Figure 3 shows |G*| and *φ* values of all tumors normalized by healthy tissue parameters (|G*|_GB_/|G*|_ref_ and *φ*_GB_/*φ*_ref_). This figure illustrates the softer tissue properties in the majority of GB and less dissipative (more elastic) properties in all tumors studied.

Additionally, lines of standard deviations are shown for the two tumors displayed in figure 2 (patients #14 und #15) in order to indicate the heterogeneity of values encountered within tumor regions. Intriguingly, standard deviations of |G*|_GB_/|G*|_ref_ do not overlap in both cases, which corroborates the visual appearance of their distinct mechanical tumor properties in Figure 2.

No correlation between tumor size and |G*| or phase angle *φ* was seen with correlation coefficients of R = −0.391 (P = 0.072) and R = 0.101 (P = 0.655), respectively. A correlation between the viscoelastic tissue parameters (|G*|, *φ*) and morphological tumor staging (Table 1) was also not seen (R = −0.235, P = 0.292 and R = −0.063, P = 0.781 respectively).

|G*| in the perifocal region was not significantly different to |G*| in GB (P = 0.306), whereas *φ* showed a significant increase in the perifocal region (P = 0.01). The significant correlation between |G*| of tumor and perifocal region (R = 0.571, P = 0.0055) indicates the extension of the tumor's viscoelastic properties into surrounding tissue.

In 16 of 22 GB included in this study, perifocal edema was visible in T2w MRI and could be outlined for MRE parameter analysis. On average, edema tissue was significantly stiffer than GB (P = 0.004), whereas *φ* was not significantly altered between tumor and edema (P = 0.99). No correlation between |G*| of tumor and edema was observed (R = 0.34, P = 0.197).

Lower values for |G*|_healthy_ at 1.5T (1.36±0.21 kPa) than at 3T field strength (1.71±0.22 kPa, P = 0.001) were observed, while none of the other parameters given in Table 2 was significantly different between 3T and 1.5T. Specifically, three of the five cases with a higher |G*|_tumor_ than |G*|_healthy_ were measured at 1.5T, which corroborates the independence of MRE parameters from MRI field strength [47].

## Discussion

Noninvasive characterization of GB remains a challenge in the present clinical routine. Conventional MRI provides only little information about tissue structure and intraparenchymal tissue connectivity. GB may include cystic, solid, and necrotic fractions as well as diffuse tumor infiltrations of the surrounding tissue; each fraction may alter the mechanical tissue properties measured by MRE.

This manuscript presents the first analysis of viscoelastic constants of intracranial tumors obtained with high spatial resolution MDEV inversion MMRE. To eliminate artifacts resulting from ill-posed inverse problems related to time-harmonic wave patterns and improve MRE parameter maps, we included multifrequency information in the solution of the inverse problem of time-harmonic elastography. The mechanical parameters elucidated in this study are well known in material science and provide full information on the complex shear modulus of human brain tissue. While |G*| relates to our haptic distinction between stiff and soft materials, *φ* represents the dispersion of the complex modulus, which is dictated by the topology of the underlying cellular network [48]. A highly elastic material such as agarose gel has a low *φ* value and is thus regarded as less dissipative than biological soft tissues composed of dense and irregular viscoelastic networks including energy-absorbing motile chains. This example illustrates the importance of considering both elastic and viscous terms for characterizing the mechanical properties of a material: agarose gel and biological tissue can have the same elasticity while their distinct viscous behavior may be appreciated by manual palpation.

|G*| and *φ* are not correlated with each other, and the two parameters convey different and independent mechanical information. In our study, this fact is illustrated by Figure 2, *φ* was clearly different in GB and healthy brain tissue, while |G*| was lower in only 17 of the 22 tumors. The uniform reduction in the dissipative GB properties may suggest a causal relationship between homeostatic tumor pressure and malignant growth as recently proposed [49] and may in the future be used as a neuroradiological marker of tumor malignancy similar to recent findings in liver tumors [50].

The heterogeneity of |G*| deserves further investigations in GB animal models. Since neither a correlation between |G*| and tumor size was observed (geometry bias) nor system-specific reasons may account for the higher stiffness in five of our patients (three were investigated at 1.5 T and two at 3 T) we expect that |G*| bears potentially valuable information for the characterization of GB. The large variability in morphological tumor assessment scores resulting from the fact that GB may be solid masses or contain cystic and necrotic fractions reflects the potential source of heterogeneity in |G*|. The fact that |G*| is not correlated to the morphological score underlines the novelty of information measured by MRE.

Although encouraging, our study has some limitations: since we conducted a pilot study, we investigated the feasibility of high-resolution MMRE in a relatively small group of patients. Future studies should include more patients and compare the findings in different tumor entities. Furthermore, no other mechanical tests could be performed to provide reference values since MRE is unique for the in vivo assessment of tumor consistency. The subjective haptic impression of surgeons in our departments varied widely, preventing us from using their scores as a gold standard of tumor consistency. Future studies in animal models can tackle this issue by using indentation tests or other microelastography methods. Finally, we cannot draw any conclusions regarding the cause of variability and the sensitivity of |G*| to diagnostically relevant tissue changes. This information has to be gathered by MRE in animal models and in a higher number of patients including post-treatment follow-up.

In summary, using multislice MMRE in combination with MDEV inversion enabled us to characterize intracranial tumors by high-resolution mechanical parameter maps of the human brain. In our cohort of 22 GB patients, the mechanical tissue parameter |G*| indicated that GB are generally softer than healthy tissue, although we noted a large heterogeneity of values. A second mechanical parameter, *φ*, which is related to the dissipative behavior of tissue, was significantly reduced in all cases. High-resolution MRE may provide an early imaging marker sensitive to pathological changes of mechanical networks in brain tissue. Its diagnostic value, in particular concerning post-treatment follow-up, has to be verified by future studies.
